# Clinical outcomes and immunological response to SARS-CoV-2 infection among people living with HIV

**DOI:** 10.3389/ebm.2024.10059

**Published:** 2024-04-02

**Authors:** Esimebia Adjovi Amegashie, Prince Asamoah, Lawrencia Emefa Ami Ativi, Mildred Adusei-Poku, Evelyn Yayra Bonney, Emmanuel Ayitey Tagoe, Elijah Paintsil, Kwasi Torpey, Osbourne Quaye

**Affiliations:** ^1^ Department of Biochemistry, Cell and Molecular Biology, West African Centre for Cell Biology of Infectious Pathogens (WACCBIP), University of Ghana, Accra, Ghana; ^2^ Department of Medical Microbiology, University of Ghana Medical School, College of Health Sciences, University of Ghana, Accra, Ghana; ^3^ Department of Virology, Noguchi Memorial Institute for Medical Research, University of Ghana, Accra, Ghana; ^4^ Department of Medical Laboratory Sciences, School of Biomedical and Allied Health Sciences, University of Ghana, Accra, Ghana; ^5^ Department of Paediatrics, Yale School of Medicine, Yale University, New Haven, CT, United States; ^6^ Department of Population, Family and Reproductive Health, School of Public Health, University of Ghana, Accra, Ghana

**Keywords:** people living with HIV, immunological response, clinical outcomes, COVID-19, HIV/SARS-CoV-2 coinfection

## Abstract

People living with HIV (PLWH) usually suffer from co-infections and co-morbidities including respiratory tract infections. SARS-CoV-2 has been reported to cause respiratory infections. There are uncertainties in the disease severity and immunological response among PLWH who are co-infected with COVID-19. This review outlines the current knowledge on the clinical outcomes and immunological response to SARS-CoV-2 among PLWH. Literature was searched in Google scholar, Scopus, PubMed, and Science Direct conforming with the Preferred Reporting Items for Systematic reviews and Meta-analyses (PRISMA) guidelines from studies published from January 2020 to June 2023. A total of 81 studies from 25 countries were identified, and RT-PCR was used in confirming COVID-19 in 80 of the studies. Fifty-seven studies assessed risk factors and clinical outcomes in HIV patients co-infected with COVID-19. Thirty-nine of the studies indicated the following factors being associated with severe outcomes in HIV/SARS-CoV-2: older age, the male sex, African American race, smoking, obesity, cardiovascular diseases, low CD4^+^ count, high viral load, tuberculosis, high levels of inflammatory markers, chronic kidney disease, hypertension, diabetes, interruption, and delayed initiation of ART. The severe outcomes are patients’ hospitalization, admission at intensive care unit, mechanical ventilation, and death. Twenty (20) studies, however, reported no difference in clinical presentation among co-infected compared to mono-infected individuals. Immune response to SARS-CoV-2 infection was investigated in 25 studies, with some of the studies reporting high levels of inflammatory markers, T cell exhaustion and lower positive conversion rate of IgG in PLWH. There is scanty information on the cytokines that predisposes to severity among HIV/SARS-CoV-2 co-infected individuals on combined ART. More research work should be carried out to validate co-infection-related cytokines and/or immune markers to SARS-CoV-2 among PLWH.

## Impact statement

People living with HIV often experience co-infections and co-morbidities, including respiratory tract infections. SARS-CoV-2 which is known to cause severe respiratory tract infections, has been reported among PLWH. There are, however, conflicting reports on HIV patients co-infected with SARS-CoV-2 with scanty information on other human coronaviruses. Studies that reported on clinical outcomes and immunological responses were reviewed through search engines and PRISMA selection criteria, with most studies indicating similar risk factors that predisposes to disease severity. High levels of inflammatory markers, T cell exhaustion and lower positive conversion rate of IgG were identified in individuals co-infected with HIV/SARS-CoV-2. Research on cytokines and immune markers in HIV/SARS-CoV-2 co-infected individuals on combined ART is limited and therefore, necessitating further validation.

## Introduction

People living with HIV (PLWH) usually suffer from co-infections and co-morbidities including respiratory tract infections, renal impairment, hypertension, diabetes, obesity, hyperlipidemia, chronic viral hepatitis, and non-AIDS-defining malignancies among others [1, 2]. These co-infections and co-morbidities tend to limit the efficacy of the antiretroviral therapy [3]. Respiratory tract infections are of a major concern due to the compromised immune state of PLWH that makes them vulnerable to severe diseases [4].

In 2019, the novel SARS-CoV-2, a new coronavirus broke out in China, also known as (COVID-19). As of September 2023, WHO reported 770,875,433 confirmed cases of COVID-19, and 6,959,316 deaths [5] spreading throughout the globe. SARS-CoV-2 has been reported to also cause more severe RTIs in HIV patients [6, 7]. Co-infections in humans have become a topic among researchers with wide interest to know their clinical importance [8, 9].

PLWH infected with COVID-19, are thought to have more complicated clinical presentations due to immunodeficiency and immune imbalance [6]. Research has reported COVID-19 in PLWH to be severe [10]. Other studies however, indicated similarity in prevalence and deleterious outcomes among both the co-infected and mono-infected [11, 12]. Bhaskaran and others reported an increased COVID-19 mortality and morbidity risk among PLWH [13], but other researchers were not convinced about this assertion and cautioned its authenticity [14].

T cell immune activation and some cytokines play a role in HIV progression [15]. COVID-19 infection has also been investigated to be associated with some immune profiles [16]. This usually leads to a cytokine storm where cytokines are then released to control inflammation causing more white blood cells to accumulate, creating a cycle of inflammation thereby damaging the lung cells. This indicates that co-infections of these HIV/SARS-CoV-2 conditions among humans may lead to harmful immunological response and a poor prognosis of disease.

This review paper sought to outline the current knowledge of clinical outcomes and immunological response to SARS-CoV-2 infection among PLWH. It also sought to identify gaps in relation to this coinfection study.

## Materials and methods

### Selection criteria

All studies reporting on clinical outcomes and immune response among PLWH co-infected with SARS-CoV-2 were included. All immunological studies with observational studies, cohort studies, case reports, randomized controlled trials, and case series were also included. All studies meeting the above stated criteria, published from January 2020 to June 2023 and in the English language were included. Studies that do not address clinical outcomes and immunological response among PLWH co-infected with COVID-19 were excluded from this review. Letters to editors, editorials, commentaries, and brief reports that did not report on any clinical data were excluded. Literature reviews, systematic review and meta-analysis data were excluded.

### Data sources and search strategy

We searched in PubMed, Google scholar, Scopus, and Science Direct using relevant terms such as “SARS-CoV-2” or “COVID-19” and ‘HIV or “Human Immunodeficiency Syndrome” or AIDS or “Acquired Immunodeficiency Disease” or PLWH or “People Living With HIV.” We then applied extra filters to access articles on “Immunological Response” or “Immune Characterization” or “Immunological Profiles” or “T-Cell Activation and Cytokines Profiles” and “Outcomes.” Also, eligible studies were identified by scanning references by manual search.

### Study selection

Titles were imported into Endnotes for every search, and duplicates were eliminated. Titles and abstracts were used by two researchers to independently check records for eligibility. The complete texts of any publications that were thought to be possibly eligible were then retrieved, evaluated, and unanimously chosen to be included in the study. Conflicts were arbitrated by a third investigator or resolved by consensus.

### Data extraction and synthesis

Extracted data were imputed into a table. All data were in English language. Studies were curated by sampling date, study design, study place, study participants, assay type, additional tests, author, and year of publication.

## Results

**FIGURE 1:**
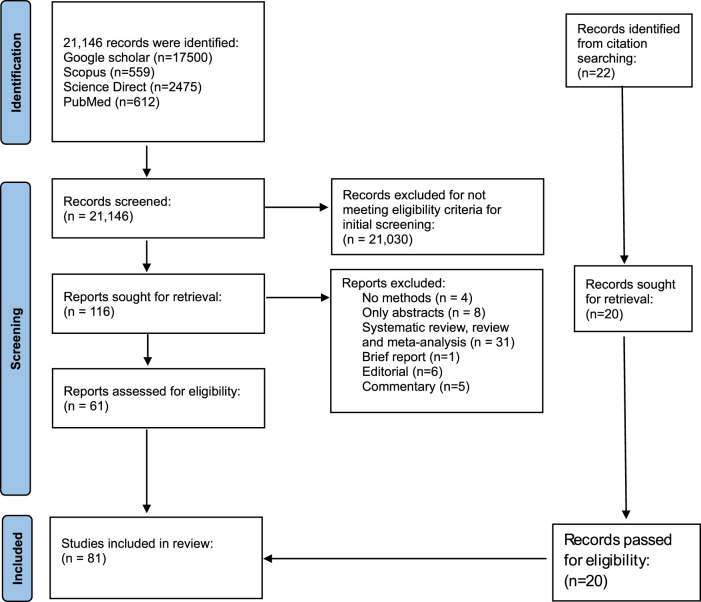
PRISMA flow diagram showing the selection criteria of studies.

Studies selection was done using PRISMA guidelines (Figure 1). Databases searches (Google scholar: 17,500, Scopus: 559, ScienceDirect: 2,475, PubMed: 612) identified a total of 21,146. Eighty-three (81) studies met the eligibility criteria after the selection process (Figure 1). Endnote Software was used to remove duplicates. Also, studies that did not meet the eligibility criteria in the initial screening were 21,031. Fifty-five (55) studies were excluded due to the following reasons: No methods (4), only abstract (8), systematic review, literature review, meta-analysis (3), Brief report (1), editorial (6) and commentary (5). A manual scanning of references resulted in 22 additional reports. Eighty-one studies were included in this analysis.

### Characteristics of included studies

Studies were identified in 25 countries in this review with United State of America contributing 17 of the included studies. Other countries where studies were carried out included South Africa (12), Italy (10), China (9), Spain (7), United Kingdom (5), France (3), Russia (2) and Brazil (2). Only one study was identified in the following countries (Korea, UAE, Iran, Germany, Japan, Guinea Bissau, Netherlands, Taiwan, Sweden, Israel, Belgium, Zambia, and Indonesia). Study participants included HIV patients, HIV naïve groups, COVID-19 patients. All studies were conducted among HIV patients. RT-PCR was used in confirming human coronaviruses in 99% of the studies. ELISA techniques were also used in 14 of the studies, followed by flow cytometry (*n* = 9), neutralization assay (*n* = 5), ELISpot (*n* = 4).

Fifty-seven studies have assessed clinical outcomes in HIV patients that were co-infected with SARS-CoV-2 (Table 1). Immune response to COVID-19 infection was investigated by 25 studies (Table 2). 18 studies were made up of brief reports, case reports and editorials with clinical and laboratory data. All the studies were carried out from 2020 to 2023.

**TABLE 1:** Summary of clinical outcomes on HIV and SARS-CoV-2 co-infection studies and their spatial distribution.

Sample date	Study design	Study place	Study participants/Sample size	Assay type	Additional tests	Key findings/Outcomes	Limitations	References
2nd March-15th April 2020	brief report	United States	72 HIV patients	RT-PCR	Viral load, CD4^+^ count, IL6, CRP, IL8, fibrinogen, D-dimer, TNF, IL-1B	High inflammatory markers and immune dysregulation linked to death in PLWH.	Study was a retrospective record limited to 1 hospital system. Complete HIV history was not available on all patients, and laboratory markers were obtained at the discretion of treating physicians	[17]
21st February-15th April 2020	Case report	Italy	383 COVID-19 patients	RT-PCR	Viral load, CD4^+^ count, FBC, LFT, CRP, procalcitonin, Chest Radiograph	Not Reported	Small sample size	[59]
			2 HIV co-infected					
1st March-7th June 2020	cohort study	United States	2988 HIV participants	RT-PCR	Viral load	PLWH were more prone to hospitalization or death as compared to non-PLWH.	Analyses were limited to demographic and laboratory data available in registry and COVID-19 database, with inadequate information on co-morbidities and underlying medical conditions	[60]
1st April-1st July 2020	Non random sampling	United States	286 HIV participants	RT-PCR	Viral load, CD4^+^ count	Older age, chronic lung disease, low CD4^+^ count, and hypertension were associated with mortality	COVID-19 testing, treatment, and hospitalization were all done at the discretion of individual healthcare providers. There may be selection bias due to error in entries of data	[61]
1st February-15th April 2020	Observational cohort study	Spain	77,590 HIV+ patients	RT-PCR		PLWH on TDF/FTC treatment have lower risk of diagnosis and hospitalization compared to those on other ART regimen	Confounding by individual clinical characteristics cannot completely explain lower risk of COVID-19 diagnosis and hospitalization among HIV-positive individuals receiving TDF/FTC.	[10]
			236 confirmed positive for COVID-19, 151 hospitalized					
Dec, 2020	Retrospective cohort study	England	17,282,905 adults, 27,480 HIV +, 14,882 COVID-19 death, 25 with HIV+	RT-PCR	Glucose and HbAic measurement, Renal Function test	Deprivation, ethnicity, smoking, and obesity were linked to high risk of COVID-19 death	There were no available routinely collected data on injection drug use, occupation, or contact patterns	[13]
March-April 2020	Case Report	Italy	26 HIV PATIENTS	RT-PCR	Viral load, CD4+count, FBC, CRP, Oxygen saturation, LDH	No death among PLWH co-infected with COVID-19	Small sample size	[8]
8th March-23rd April 2020	Observational Cohort study	United States	530 COVID-19, 20 PWLH	RT-PCR	Viral load, CD4^+^ count, Oxygen saturation, X-ray, CT scan	Co-morbidities among PLWH linked to COVID-19 deaths	Smaller sample size among the co-infected	[7]
21st February-16th April 2020	Retrospective study	Italy	47 HIV patients	RT-PCR	x-ray, CT scan, Oxygen saturation, Viral load, CD4^+^ count	45 PLWH fully recovered and 2 died	Not all the patients with HIV were confirmed to have COVID-19, and therefore limited the number of co-infected individual	[62]
23rd January-31st March 2020	Case series	China	12 HIV patients	RT-PCR	CD4^+^ count, Viral load	ART naïve patients presented with severe symptoms, and therefore had longer hospitalization	Study underestimated the proportion of serious cases among PLWH co-infected with COVID-19	[63]
1st January-16th April 2020	Retrospective study	China	6001 PLWH, 35 coinfected with COVID-19	RT-PCR, Magnetic Chemiluminescence Enzyme Immunoassay	Viral load, CD4^+^ count	15 HIV/SARS-CoV-2 co-infected patients had severe illness with 2 deaths. Older age and discontinued cART were associated with severe illness and death	Results from the small number of HIV/SARS-CoV-2 coinfected cannot be generalized to the population	[35]
3rd March-15th May 2020	Retrospective cohort study	United States	30 HIV patients, 90 control groups without HIV	RT-PCR	Viral load, CD4^+^ count, Lymphocyte count, LDH, D-dimer, Procalcitonin, CRP	No difference in the need for mechanical ventilation during hospitalization, length of stay or mortality between PLWH and non-PLWH who are co-infected with COVID-19	Small sample size	[64]
March-April 2020	Retrospective study	United Kingdom	18 PLWH, 16 + with COVID-19	RT-PCR	Viral load, CD4^+^ count	3 out of 18 PLWH died	Small sample size	[65]
April 10, 2020	Case Report	South Africa	2 HIV+/COVID-19+	RT-PCR	Oxygen saturation level, Arterial blood gas, X-ray, CT scan, Viral load, CD4^+^ scan	PLWH have a good outcome due to their impaired immune response	Small sample size	[66]
1st March-30th June 2020	Retrospective study	Italy	31 HIV+/COVID-19+	RT-PCR	Viral load, CD4^+^ count, Oxygen saturation level	Patients did not require ventilation and recovered 9 days after admission	Small sample size	[67]
Not stated	Case report	United Arab Emirates	1 HIV+/COVID-19+, Kaposi +	RT-PCR	FBC, Viral load, T cell differential count, Renal function test, Blood culture, Respiratory pathogen panel, LDH, D-dimer, Fibrinogen, Ferritin Procalcitonin, CRP	Co-morbidities were associated with death	Small sample size	[68]
12th March-23rd April 2020	Cohort study	United States, New York	4402 COVID-19+, 88 PLWH	RT-PCR	White blood count, creatinine, ALT, ferritin, IL-6, D-dimer, LDH, Procalcitonin, CRP, oxygen saturation level	There was frequent hospitalization among PLWH compared to non-PLWH.	Small sample size of PLWH	[11]
20th March-30th April 2020	Retrospective study	United States	14 PLWH coinfected with COVID-19	RT-PCR	X-ray, Viral load	8 patients were hospitalized and 6 self-quarantined. There was no death	Small sample size	[69]
January-April 2020	Cohort study	France	30 HIV patients, 90 control groups without HIV	RT-PCR	CD4^+^ count, Viral load	80% recovered from COVID-19 infection, 10% required ventilation, 6.7% died and 13.3% required hospitalization	Not reported	[70]
January-April 2020	Observational prospective study	Spain	51 HIV patients	RT-PCR	Viral load, CD4^+^ count, Full blood count, Renal function test, ALT, Procalcitonin, CRP, ferritin, IL-6, IL-12, LDH, D-dimer, X-ray, Oxygen saturation level	4% of the HIV/SARS-CoV-2 individuals died	The small number of individuals prevented generalisation of results	[6]
8th February 2020	Case report	China	2 HIV patients	RT-PCR	IL-6, procalcitonin, ferritin, CRP, Albumin, CD4^+^ count, Viral load, X-ray, Sars CoV2 abs test	Patients recovered.	Results were based on only two patients, and no follow-up was done due to limit resource	[71]
2nd March-23rd April 2020	Retrospective study	United States, New York	21 HIV+, 2617 HIV- COVID-19+	RT-PCR	FBC, procalcitonin, CRP, Troponin, D-dimer, ferritin, LDH, Creatinine, Creatinine phosphokinase, Respiratory rate, CD4^+^ count, Temperature, Blood pressure	Co-morbidity, higher inflammatory markers were associated with higher admission. All patients with comorbidity died	Lack of clinical data on participants	[72]
							Small sample size	
11th March-17th April 2020	Retrospective study	Germany	33 HIV participants	RT-PCR	Respiratory rate, CD4^+^ count, Viral load, Oxygen saturation	3 of the patients died, 91% recovered and 76% mild cases	Small uncontrolled case series with limited follow up	[73]
January-9th March 2020	Case series	Spain	543 COVID-19 patients	RT-PCR	Viral load, CD4^+^ count, oxygen saturation, CRP, LDH, D-dimer, FBC	4 out of 5 patients were cured by the end of the study	Small sample size	[74]
			5 HIV patients: 3 MSM, 2 transgenders					
15th March-15th April 2020	Case report	United States, New York	31 PLWH infected with COVID-19	RT-PCR	Vural load, CD4^+^ count, CRP, D-dimer, Ferritin, Procalcitonin, radiological findings	8 patients died, and 21 recovered	Smaller sample size	[75]
							There was no comparison with patients without HIV.	
March 2020-September 2021	Case series	Tokyo, Japan	17 HIV-COVID-19 patients	RT-PCR	CD4^+^ count, lymphocyte, CD8^+^ count	All patients recovered. No difference in CD4^+^ and CD8^+^ counts between onset of symptoms and after recovery	Small sample size	[28]
2nd January–31st October 2020	Observational retrospective monocentric cohort	Paris, France	129 HIV individuals with COVID-19	RT-PCR	Viral load, CD4^+^ count	Older age, higher BMI, diabetes, chronic kidney disease, transgender women were prone to disease severity with poor outcomes	Not all patients were confirmed to have COVID-19 by PCR.	[76]
January 2020- Not stated	Retrospective cohort study	Italy, Rome	1647 hospitalized patients, 43 PLWH, 1605 non PLWH	RT-PCR	CD4^+^ count, Full blood count, Viral load, Potassium, CRP, D-dimer, Ferritin, oxygen saturation	There was less death among PLWH as compared to non-PLWH.	Small sample size	[77]
							Analysis done at the study site cannot be generalized to other sites	
29th March 2020	Case Report	Korea	1 HIV positive man	RT-PCR	CD4^+^ and CD8^+^ count, Viral load, Chest X-ray, CT scan, ESR and platelet count	Patient recovered	small sample size	[78]
May 2020	Case Report	Brazil	1 HIV positive woman	RT-PCR	CD4^+^ count, CD8^+^ count, Viral load, Chest X-ray, Oxygen saturation level	Patient recovered after a week	Small sample size	[79]
1st march–9th June 2020	Cohort study	Cape Town, South Africa	3,460,932 public patients, 3978 HIV patients with COVID-19	RT-PCR	CD4^+^ count, CD8^+^ count, Viral load	HIV and tuberculosis were associated with COVID-19 mortality	There was lack of data on co-morbidities and potential risk factors	[80]
11th June–28th August 2020	Observational case control study	Cape-Town, South Africa	104 COVID-19 positive patients, 31 HIV/COVID-19 patients	RT-PCR, Neutralization assay, Flow cytometry	Cd4+ count, Viral load, LDH, Ferritin, D-dimer	30 patients died	Analysis was not empowered to reproduce relationships between HIV and severity of COVID-19	[81]
1st June–1st October 2021	Observation study	Guinea Bissau	294 PLWH	COVID-19 IgM/IgG rapid test kit	-	Six deaths reported	The study population consisted only of patients on follow-up	[82]
			55 PLWH positive for SARS-CoV-2					
1st March–30th April 2020	Observational prospective monocentric study	France	54 PLWH coinfected with COVID-19	RT-PCR	Viral load, CD4^+^ count, IL-6	Male sex, age, ethnic origin, metabolic disorder was associated with severity of disease. 2 deaths were recorded	Study did not assess the risk linked to immune deficiency	[83]
17th January–18^th^ June 2020	Prospective Observational study	United Kingdom	115 HIV patients	RT-PCR	Full blood count, prothrombin time, Creatinine, CRP	63% increased risk of day 28 mortality among PLWH hospitalized with COVID-19 compared to HIV negative	Risk factors for a COVID-19 related hospitalisation among PLWH, and the role of certain antiretroviral agents in modulating such risks were not addressed	[84]
			47,424 HIV negative patient					
Not stated	Prospective study	China	1178 HIV patients, 8 co-infected	RT-PCR	CD4^+^ count, Viral loads, CT scan	Older ages were prone to get infected with COVID-19	Small co-infection size	[85]
Not stated	Retrospective cohort study	Massachusetts, United States	49,673 non PLWH, 404 PLWH	RT-PCR	CRP, LDH, ALT, AST, Bilirubin, Ferritin	PLWH had higher mortality at day 30 and were likely to be hospitalized that non PLWH.	Lack of clinical data	[86]
9th March 2020–8th March 2020	Retrospective study	Burkina Faso	419 PLWH	RT-PCR, ELISA		PLWH on integrase inhibitors were more likely to be infected than PLWH on non-nucleoside inhibitors	Study could not investigate if COVID-19 natural infection may confer comparable antibody immunity among PLWH.	[87]
June 2020–May 2021	Prospective Cohort study	South Africa	236 PLWH, 143 non-PLWH	RT-PCR. Flow cytometry	CD4^+^ and CD8^+^ count, Viral load, Full blood count	Higher disease severity was associated with low CD4+count and higher Neutrophil to lymphocyte ratio in first wave as compared to second wave	Limited information on HIV related immune perturbations influencing long-term immunity to SARS-CoV-2 infection	[88]
Until March 2022	Cross-sectional study	South Africa	600,00 PLWH	RT-PCR	CD4+count, Viral load	Mortality occurred in 5.7% of PLWH. Mortality was associated with lower recent CD4^+^ count, no evidence of ART usage, high viral load, and co-morbidities	Study did not assess the impact of prior SARS-CoV-2 infection on COVID-19 outcomes	[89]
Not stated	Case Report	Netherlands	A 38-year-old male HIV patient	RT-PCR	CD4^+^ count, Viral load, Chest X-ray	Patient was admitted with prolong COVID-19 infection with undiagnosed HIV and severe impaired cellular immunity	Small sample size	[90]
1st March 2020–30th November, 2020	Prospective Cohort study	United States	1785 PLWH, 189,350 non-PLWH	RT-PCR	Viral load, CD4^+^ count	15% were hospitalized and 5% died. Tenofovir was associated with reduction in clinical events	Results covered the first 9 months of the pandemic and did not include follow up during second wave	[36]
1st June 2020–15th June 2020	Prospective cohort study	Italy	55 COVID-19 positive patients, 69 HIV patients negative for COVID-19	RT-PCR, ELISA	CD4 and CD8^+^ counts, Viral load, Chest X-ray	4 deaths were recorded	Small sample size prevents generalization of results	[91]
						Age and number of co-morbidities were associated with death		
Not stated	Case report	New York, United States	A 54-year-old man	RT-PCR	CD4^+^ count, Biochemical test, Coagulation profile, Ferritin, Il-6, Procalcitonin, CK-MB, Chest X-ray	Patient recovered	Small sample size	[92]
February 2020–October 2021	Retrospective study	Sweden	64, 815 COVID-19 patients: 121 HIV positive	RT-PCR	Viral load, CD4^+^ count	8% of PLWH died. HIV infection was not a risk factor for severe COVID-19	Number of hospitalized PLWH were small therefore limited the power	[93]
1st January 2020–31st December, 2020	Retrospective study	Spain	117,694 COVID-19: 234 HIV positive	RT-PCR	Comorbidity assessment	9.4% mortality among PLWH. Advanced liver disease was a predictor of death	Overestimation of hospitalization among PLWH.	[94]
10th March 2020–30th May 2020	Retrospective cohort study	Israel	23 PLWH coinfected with COVID-19	RT-PCR	Full blood count, Viral load, CD4^+^ count	13% of in-hospital death, 9% mechanical ventilation, and 9% intensive care unit admission were recorded	Small sample size	[95]
15th February–31st May 2020	Retrospective multicentre cohort study	Belgium	16,000 HIV patients:101 COVID-19 patients	RT-PCR	CD4^+^ count, Viral load, CT scan	46% of patients were hospitalized, and 9% of patients died. Older age, sub-Saharan patients and those on integrase inhibitor were associated with hospitalization	Small sample size	[96]
							No comparison made with non-HIV patient	
1st March 2020–15th December 2020	Observational prospective cohort study	Spain	13,142 followed up HIV patients: 749 COVID-19 positive	RT-PCR	Viral load, CD4^+^ and CD8^+^ count	13 patients died. Chronic co-morbidities were associated with severe outcomes	Not all cohorts were tested for SARS-CoV-2 so incidence rate was not assessed. Data did not include information on smoking and BMI.	[97]
December 2021	Brief report	South Africa	45-year-old man	RT-PCR, Viral sequencing	Viral load, CD4^+^ count, Chest x-ray, IgG and IgA antibodies	Prolonged infection in HIV individuals may lead to evolution of SARS-CoV-2 lineages	Not reported	[98]
10th March 2020–6th June 2020	Retrospective matched cohort study	New York, United States	853 PLWH and 1621 HIV negative patients	RT-PCR	Viral load, CD4^+^ count, FBC, Biochemical test, CRP, D-dimer, Ferritin, Procalcitonin, ESR, fibrinogen	Hospitalized PLWH and controls show no difference in-hospital death. Co-morbidities and inflammatory markers differ for each cohort upon hospitalization indicating different mechanisms leading to severe COVID-19	Data from Medical record did not include a history of treatment for co-morbidities, which prevented accounting for severity or progression of these conditions	[99]
March 2020–September 2020	Observation Retrospective cohort study	Brazil	17,101 COVID-19 patients: 130 PLWH	R- PCR	Viral load, CD4^+^ count, full blood count, Kidney function test, Liver function test, Arterial pH, pO_2,_ pCO_2_	27.9% mortality in PLWH in 2020. No difference in mortality among PLWH and non-PLWH in 2021	Small sample size	[23]
March 2021–December 2021								
1st January 2020–21st May 2021	Retrospective cohort study	United States	1,446,913 COVID-19 patients: 3660 PLWH	RT-PCR	Viral load, CD4^+^ count	PLWH with immune dysfunction have greater risk for severe COVID-19 outcomes	There was less representation of admission practices and disease severity in medical records from study site	[100]
1st March 2020–30th November 2020	Retrospective cohort study	United States	487 COVID-19 patients: 88 PLWH	RT-PCR	Viral load, CD4^+^ count	People living with HIV have a higher risk of COVID-19 diagnosis than those without HIV, but the outcomes are similar in both groups	Study could not implement routine SARS-CoV-2 screening to identify all patients with SARS-CoV-2 infection	[101]
1st January 2020–8th May 2021	Retrospective cohort study	United States	1, 436, 622 COVID-19 patients: 13, 170 HIV patients	RT-PCR	CD4^+^ count, Viral load	A low CD4^+^ count was associated with all the adverse COVID-19 outcomes, while viral suppression was only associated with reduced hospitalisation	Though was a large sample size, this did not represent all the COVID-19 infections in the country	[102]
1st March–31st December 2020	Retrospective cohort study	Indonesia	4134 PLWH	RT-PCR	Viral load, CD4^+^ count	23 patients developed severe-critical COVID-19, and the mortality rate was 3.2%	The incidence of infection in the study population could not be assessed because not all participants were tested for SARS-CoV-2	[103]
			342 PLWH with COVID-19					
28th January 2020	Editorial	China	61-year-old HIV man	RT-PCR	FBC, oxygen saturation, CT scan, CD4^+^ count	HIV infection need to be regarded as vulnerable group	Small sample size	[104]

**TABLE 2:** Summary of immunological response on HIV and SARS-CoV-2 co-infection studies and their spatial distribution.

Sampling date	Study design	Study place	Study participants/Sample size	Assay type	Additional tests	Clinical outcomes/key findings	Limitations	References
2nd March-15th April 2020	brief report	United States	72 HIV patients	RT-PCR	Viral load, CD4^+^ count, IL6, CRP, IL8, fibrinogen, D-dimer, TNF, IL-1B	High inflammatory markers and immune dysregulation were linked to death in PLWH.	Study was limited to 1 hospital. Complete HIV history was not available on all patients, and laboratory markers were obtained at the discretion of treating physicians	[17]
June-October 2020	Prospective study	Iran	155 HIV-1 patients	RT-PCR, Enzyme immunoassay	Viral load, CD4^+^ count, Hepatitis B, C, Tb test,	Higher anti-SARS-CoV-2 were reported in males than females	Screening and identification of HIV-1-infected individuals were limited due to COVID-19 lockdown	[29]
June-November 2020	Longitudinal study	South Africa	72 COVID-19 patients, 42 without HIV, 30 with HIV, 25 on ART	RT-PCR, Enzyme immunoassay (IgA, Igg, IgM), Microneutralization assay, Whole genome sequencing	CD4^+^ count, CD8^+^ count, Viral load	Antibody response among PLWH were comparable to those of non-PLWH.	Sample size small. There is a possibility of missing peak IgM response due to time of sampling	[12]
							Only 16 out of 72 full genomes were sequenced	
January-June 2020	retrospective study	Italy, Spain & Germany	175 HIV patients	RT-PCR	Viral load, CD4^+^ count	Low CD4^+^ count was associated with high mortality rate in PLWH.	Only data on absolute CD4 T cells were available. Data on other lymphocyte subpopulations such as CD8 T cells were lacking	[105]
December 2020	Case report	Spain	1 HIV patient	RT-PCR, Whole Genome sequencing, Flow cytometry	Viral load, CD4^+^ count, full blood count, Computed Topography (CT) scan	T cell exhaustion was associated with severity of disease among PLWH.	Small sample size	[18]
Not stated	Case report	Italy	1 HIV patient	RT-qPCR, flow cytometry, RT-PCR	CD4^+^ and CD8^+^ count, viral load, FBC, Arterial blood gas, Computed Topography scan	IFNα/β mRNAs and T cell activation were associated with severe pneumonia	Small sample size	[19]
15th January-20th November 2020	Prospective Cohort study	China	18 PLWH, 185Non PLWH	RT-PCR, Immunochromatography assay	Lymphocyte count	Positive conversion rate of IgG was lower and quickly lost in PLWH compared to non-PLWH.	Lack of an antibody detection kit in the early days of the SARS-CoV-2 epidemic prevented early antibody testing	[30]
20th March-15th June 2020	Cohort study	Russia	376 (171 ART experienced, 205 ART naïve)	RT-PCR, Flow cytometry, ELISA	Respiratory score	HIV ART-naïve was reported as a strong co-morbidity of severe COVID-19	Not reported	[32]
			382 control groups					
1st March-12th May 2020	Observational study	Italy	604 HIV participants	RT-PCR, Immunofluorescence, Microneutralization test, Flow cytometry, Elispot assay, ELISA	CT scan, Lymphocyte count, LDH, D-dimer, Fibrinogen, Ferritin	Adaptive cellular immune response correlated with disease severity	Small sample size makes it difficult to distinguish real effects from random variations thereby no definitive conclusions can be made	[20]
11th February 2020	Case report	China	1 HIV patient	RT-PCR	CRP, LFT, LDH, FBC, oxygen saturation	Slower generation of antibodies was attributed to severity of disease	Small sample size	[31]
6th March–11th September 2020	Retrospective study	South Africa	676 COVID-19 patients: 108 HIV	RT-PCR, ELISA	CD4^+^ count, Viral load, FBC, RFT, LFT, CRP, Troponin T, LDH, D-dimer, Troponin T, Ferritin, beta-d-glucan, procalcitonin	No significant difference in mortality between the HIV-positive and HIV- negative groups. HIV-positive patients who died were younger than the HIV-negatives	It was a single centre study, and so the data may not be generalized. some data capturing was retrospective, due to rapidly increasing patient numbers and staff shortages, and as a result some data were missing	[106]
8th February 2020	Case report	China	2 HIV patients	RT-PCR	IL-6, procalcitonin, ferritin, CRP, Albumin, CD4^+^ count, Viral load, X-ray, Sars CoV2 abs test	Patients recovered	Small sample size	[71]
8th June 2020–25th September, 2020	Cross sectional study	South Africa	126 HIV participants	RT-PCR, Flow cytometry, ELISA	CD4^+^ and CD8^+^ counts, Viral load	B cell responses were rapid but gave rise to lower affinity antibodies, less durable long-term memory, and reduced capacity to adapt to new variants	Study could not determine long-term effects of HIV on SARS-CoV-2 immunity, as new variants emerge	[27]
June–December 2020–1st wave	Longitudinal observational cohort study	South Africa	25 HIV participants 1st wave	RT-PCR, Flow cytometry	Viral load, CD4^+^ count	Unsuppressed HIV infection impaired T cell responses to SARS-CoV-2 infection and diminishes T cell cross-recognition	Study did not examine relationship between CD8^+^ and CD19 subset at antigen-specific level due to sample limitations	[26]
January–June 2021- 2nd wave			23 HIV participant 2nd wave					
			HIV negative 17					
Not stated	Case report	Taiwan	A 38-year-old man	RT-PCR, ELISA, Virus neutralization assay	CRP, Viral load, CD4^+^ count, ALT, AST, Chest X-ray	Neutralizing antibody reached a plateau from 26th to 47th day onset but decreased on 157th day after symptoms	Small sample size	[107]
4th March 2020	Cross sectional study	China	24 HIV patients: 21 had COVID-19	RT-PCR	Ct scan, Chest X-ray, CRP, Procalcitonin, Viral load, CD4^+^ count, Full blood count, coagulation profile, Biochemical test	Reduction in T-cell number positively correlates with the serum levels of interleukin 6 (IL-6) and C-reactive protein (CRP)	Small sample size. Lack of detection of TCR zeta-chain expression	[30]
Not stated	Prospective cohort study	Zambia	46 HIV negative patients	RT-PCR, Immuno-spot assay. Immunofluorescence assay, flow cytometry	CD4^+^ count, viral load	SARS-CoV-2-specific T cell immune responses may be delayed in individuals who are HIV +, even in those on antiretroviral therapy. There is no difference in SARS-CoV-2- specific humoral immunity between individuals who are HIV- and HIV+	Small sample size limited study’s ability to elicit some of the differences that might exist between the sub- groups	[33]
			39 HIV positive patients					
25th January 2020	Brief Report	China	38-year-old HIV man	RT-PCR, Chemiluminescence assay	FBC, CRP	Total Ab level was largely increased, and IgM remained at the peak level 1 week later, suggesting that the antibody responses against SARS-CoV-2 in this HIV-infected case was delayed	Studies did not to address the mechanism underlying the delayed antibody response to SARS-CoV-2 with a history of coinfection of HIV-1 infection	[108]
June 2020–August 2020	Prospective cohort study	South Africa	133 hospitalized patients: 95 COVID-19 patients (31 positive for HIV)	Flow cytometry, RT-PCR, electro chemiluminescent immunoassay	CD4^+^ count, Viral load, CRP, D-dimer, LDH, ferritin, WBC	SARS-CoV-2–specific CD4^+^ T cell attributes were associated with disease severity	Study could not use different approaches (such as the activation-induced markers assay) to confirm the inability of lymphopenia patients to mount a T cell response to SARS-CoV-2	[21]
			30 non COVID-19 patients (with 13 positives for HIV)			Severe disease was characterized by poor polyfunctional potential, reduced proliferation capacity, and enhanced HLA-DR expression		
5th May 2020–22nd February, 2021	Retrospective Cohort study	United States	2464 PLWH: 283 COVID-19 positives	RT-PCR, ELISA, Luminex assay	Viral load, CD4^+^ count	SARS-CoV-2–specific humoral immune profiles among PLWH with obesity or lower nadir CD4^+^ T cell count was associated with worse outcomes	The study’s cross-sectional nature limits the ability to assess humoral repertoire changes over time in relation to COVID-19. Study was not able to control for statin use, given the ongoing nature of the trial	[109]
May 2020–October 2020	Observational cohort study	United States, Peru	43 PLWH, 330 non PLWH	RT-PCR. ELISA	Viral load, CD4^+^ count	Decreased SARS-CoV-2–specific antibodies among PLWH compared to non PLWH.	The median duration from diagnosis to enrolment was nearly 2 months, which did not fully represent the convalescent period	[34]
						PLWH who recovered from COVID-19 had diminished immune responses and lacked an increase in SARS-CoV-2 antibodies		
Not stated	Prospective study	Barcelona, Spain	50 patients, 11 PLWH, 39 non PLWH	RT-PCR, EliSpot immune assay, Fluorospot immune assay, ELISA	Oxygen saturation	PLWH developed a comparable short and long-term natural functional cellular and humoral immune response than non PLWH convalescent patients, which are highly influenced by the clinical severity of the COVID-19 infection	Patients with critical COVID-19 (requiring Mechanical ventilation) could not be obtained during the first wave of the pandemic and may be under-represented in this study	[110]
September–November 2020	Observational cohort study	Italy	HIV with COVID-19: 30	RT-PCR, micro-neutralization assay, ELISpot assay,	Viral load, CD4^+^ count, oxygen saturation	Significantly higher levels of IL-6, IL-8, and TNF-α in COVID-19 without HIV compared to HIV/COVID-19 patients were observed	Studies did not evaluate the persistence of these immunity and its ability to expand after exposure	[24]
			HIV without COVID-19: 52					
			COVID-19 without HIV: 58					
April-September, 2020	cohort study	Italy	Young HIV patients	RT-qPCR, ELISA, Magnetic bead immunoassay, Geneplex assay, cytokine multiplex assay	CD4^+^ count, Liver function test, Renal function test, Clotting Profile	IL-10 could play a crucial role in the course of SARS-CoV-2 infection in HIV-positive individuals	Small sample size could lead to higher variability	[52]
			85 ART experienced control group 13					
March 2020–September 2021	Cohort study	United Kingdom	47 HIV individuals, 24 confirmed COVID-19, 35 HIV negative	RT-PCR	Lymphocyte count, CD4^+^ count, CD8^+^ count, Spike IgG, N IgG antibodies, IFN-γ, TNF-α	Inadequate immune reconstitution on ART, could hinder immune response to SARS-CoV-2	Study was not well powered	[25]

Legend: PLWH, people living with HIV; HIV, human immunodeficiency virus; CRP, C-reactive protein; FBC, full blood count; TNF, Tumour Necrotic factor; LFT, liver function test; RFT, renal function test; IL, interleukin; LDH, lactate dehydrogenase; RT PCR, Real time polymerase chain reaction; IgG, Immunoglobulin G; ALT, alanine transferase; AST, Aspartate Transferase; CT, scan, Computed topography scan; ESR, erythrocyte sedimentation rate; WBC, white blood cells; pCO2, Partial pressure of carbon dioxide; pO2, partial pressure of oxygen.

Thirty (38) studies reported the following risk factors as associated with severity of diseases (Table 1). This includes older age, higher BMI, male sex, deprivation, ethnicity, obesity, smoking, Tuberculosis, chronic kidney disease, higher inflammatory markers, diabetes, cardiovascular disease, lung cancer, African American, high viral load, low CD4^+^ count, high neutrophil-lymphocyte ratio, discontinued ART usage and some ART regimen. Twenty studies however indicated that clinical presentations among the co-infected were the same as the general population therefore there was low risk of disease severity (Table 1).

Twenty-five studies looked at immunological responses (Table 2), out of which four suggested that high inflammatory markers and immune dysregulation are linked to severity of disease and death among people who are coinfected with HIV/SARS-CoV-2 and are on ART, even though the ART is supposed to help with HIV viral suppression and immune reconstitution [17–22]. HIV/SARS-CoV-2 individuals with higher pro-inflammatory markers such as C-reactive protein (CRP), IL-8, IL-6 presented with disease severity and higher mortality than those who recovered [17]. Three other studies on co-infections linked reduction of T cell numbers to increased IL-6, IL-8, and CRP levels, causing a cytokine storm [23–25]. Among the co-infected individuals, unsuppressed HIV hampers T cell cross-recognition and responses to SARS-CoV-2 infection, and thereby leading to severe outcomes [26, 27]. The pre-symptom and post recovery CD4^+^, and CD8^+^ counts showed no significant difference between PLWH and HIV negative individuals who are infected with SARS-CoV-2 [28]. PLWH saw a brief decline in CD4^+^, and CD8^+^ counts during the acute phase of COVID-19 with the CD4+/CD8+ ratio remaining unchanged [11, 28].

Most of the studies were either retrospective or prospective with one time point sample collection, therefore, no subsequent CD4^+^ counts and viral loads to determine relationship with clinical outcomes. Two of the studies were longitudinal with one study investigating two waves of SARS-CoV-2 infection [26] and the other following up for a period of 3 months on HIV/SARS-CoV-2 patients [12]. Snyman *et al.,* indicated in their study that anti-SARS-CoV-2 IgM, IgG, and IgA levels in non-HIV individuals and PLWH on full HIV suppression on ART have similar seroconversion rates [12]. The conversion rate of anti-SARS-CoV-2 IgG was lower and quickly lost in PLWH as compared to HIV negative persons who are SARS-CoV-2 positive [29–31]. Three of the studies indicated that slower generation of anti SARS CoV2 antibodies were attributed to increased COVID-19 severity among PLWH [32–34].

## Discussion

We conducted a scoping review to assess specific COVID-19 clinical outcomes and immune response in patients with human immunodeficiency virus (PLWH) and identify gaps. Hospitalisation risk, intensive care unit admission, mechanical ventilation and mortality were the four categories identified as clinical outcomes. Our review showed varied reports on risk of hospitalisation, ICU admission, mechanical ventilation and mortality in cohort studies, case series, and case reports. PLWH who died exhibited higher levels of soluble immune activation and inflammation markers, which are linked to disease severity in COVID-19 [22]. Individuals with non-suppressed HIV viremia have reported lower levels of antibodies against SARS-CoV-2 in their humoral response [35]. Some studies however, associated low risk of hospitalization and death to Tenofovir usage as compared to those on other regimen [35–37].

### Immune response to SARS-CoV-2 infection among PLWH on ART

ART does not eradicate HIV completely but significantly reduces morbidity and mortality associated with the virus [38]. ART may also reduce the severity of COVID-19 through immunological reconstitution, although these effects have not yet been confirmed [10, 36, 39]. PLWH with mild COVID-19 presentation, in the presence of high proinflammatory markers, suggested that certain antiretroviral drugs were protective against severity of COVID-19 disease [20]. A study in Russia among 376 HIV/COVID-19 patients (171 without ART and 205 with ART) suggested that elevated anti-inflammatory markers such as IL-10 and TGFβ, reduced CD4+/CD8+ cell ratios led to an increase in exhausted T cells in ART naïve patients. This led to Adverse Respiratory Distress Syndrome among the ART naïve group [32]. Sharov also reiterated that in the presence of uninterrupted ART, HIV patients do not progress to severe SARS-CoV-2 infection [32]. Other studies hypothesized that specific ART (NRTIs, NNRTIs and PI) predisposes to severe COVID-19 but no conclusive findings have been made because of studies involving smaller sample size and inconsistent cases and reports [40, 41].

### Signaling pathway of HIV/SARS-CoV-2 coinfection

Viral infections interact mainly with the activated Signal Transducer and Activators of Transcription 1, 2, and 3 (STAT1, STAT2 and STAT3) to release pro-inflammatory cytokines to eliminate viruses [42]. The IL-6-JAK-STAT3 axis is significantly linked to the onset of severe COVID-19 [43, 44]. The dimerized epidermal growth factor receptor (EGFR) can tyrosine-phosphorylate STAT3, which is elevated in cases of acute lung injury [45] and in cases where STAT1 is lacking [46, 47]. As a result, in COVID-19, EGFR signalling may develop into a different pathway that stimulates STAT3 when lung damage occurs, and SARS-CoV-2 infection significantly reduces IFN-I production [48]. This aberrant transcriptional rewiring towards STAT3 may lead to the symptoms most typically reported in hospitalised COVID-19 patients: fast coagulopathy/thrombosis, proinflammatory conditions, profibrotic state, and T cell lymphopenia [49].

Some HIV proteins have been reported to inhibit effective IFNα signalling by degrading certain components of the JAK/STAT signalling pathway like STAT1 and STAT3 [50]. The impaired JAK/STAT signalling pathway is however restored in the presence of uninterrupted combined Antiretroviral therapy (cART) for more than 6 months [51]. Per our search, we found one study available on HIV/COVID-19 signalling pathway that investigated STAT3 but did not look at other STAT pathways [52], and therefore creates a gap that needs to be researched. Understanding the viral co-infection, immune response, and signalling pathway dynamics will help identify particulate markers that predisposes to severity of disease.

### Oxidative stress responses among HIV/SARS-CoV-2 coinfection

Hyperactivation of STAT3 affect various biological and physiological functions, leading to oxidative stress (OxS) and poor prognosis of disease [22]. Oxidative stress (OxS) comes about by accumulating reactive oxygen and nitrogen species, which are free radicals that causes injury to organs. Under physiological conditions, these OxS are wiped out by antioxidants especially glutathione (GSH) [53]. Glutathione are endogenous intracellular antioxidants that neutralizes free radical released due to oxidative stress [54]. Deficiency in GSH however, leads to high levels of OxS due to compromised antioxidant defences [55]. Oxidative stress has been studied in HIV or SARS-CoV 2 alone with higher levels reported in each disease [55–58]. There is however scanty information on oxidative stress among HIV/COVID-19 patients, hence the need to investigate if the presence of ART usage affects oxidative stress response.

### Limitations

There is lack of information on cellular immunity in other hCoVs apart from COVID-19 co-infection. Cytokine have been studied extensively in HIV or COVID-19 alone but not as a co-infection. The oxidative stress levels among HIV/SARS-CoV-2 co-infection are yet to be studied although research has been done for other co-morbidities or co-infections.

## Conclusion

This study highlights the paucity of clinical and immunological data on HIV/SARS-CoV-2 co-infection in sub-Saharan Africa, even though this region has the highest HIV prevalence. Review shows conflicting reports on severity of the co-infection. HIV/SARS-CoV-2 severity and outcomes appear to be worse, when coexisting age-related comorbidities and CD4 + T-cell depletion is present. Discontinued or no evidence of ART usage have also been shown to increase disease severity, which needs to be studied further to ascertain its authenticity.

CD4^+^ T cell lymphopenia in both diseases is influenced by various mechanisms including direct attacks, immune activation, and redistribution of CD4^+^ T cells. Cytokines investigation will help identify markers that are implicated in disease severity among HIV/SARS-CoV-2 patients. Further investigation is needed to confirm co-infection-associated cytokines and/or immunological markers to SARS-CoV-2 in PLWH.

## Data Availability

The original contributions presented in the study are included in the article/supplementary material, further inquiries can be directed to the corresponding author.
